# Reply to Zhu et al.: Implications of *CHRNB1* and *ERBB2* in the pathobiology of myasthenia gravis

**DOI:** 10.1073/pnas.2209096119

**Published:** 2022-08-15

**Authors:** Ruth Chia, Sara Saez-Atienzar, Daniel B. Drachman, Bryan J. Traynor

**Affiliations:** ^a^Neuromuscular Diseases Research Section, Laboratory of Neurogenetics, National Institute on Aging, Bethesda, MD 20892;; ^b^Department of Neurology, Johns Hopkins School of Medicine, Baltimore, MD 21287;; ^c^Reta Lila Weston Institute, UCL Queen Square Institute of Neurology, University College London, London WC1N 1PJ, United Kingdom;; ^d^National Institute of Neurological Disorders and Stroke, Bethesda, MD 20892

We commend the work performed by Zhu et al. providing additional insights into the pathogenesis of myasthenia gravis (1). In essence, they applied Mendelian randomization to genome-wide data that we recently made public for a large cohort of patients diagnosed with the neuromuscular disorder (2). This powerful approach identified genetic variants increasing the risk of developing myasthenia gravis by influencing gene expression. Their most exciting observations centered on *CHRNB1* and *ERBB2*, two loci we discovered in our genomic and transcriptomic analyses (2). Their data corroborated our conclusions in that they found rs4151121 to influence the expression of *CHRNB1* in skeletal muscle. *ERBB2* was also implicated in their search, though the lead variant and tissue involved differed between the two studies. We had identified rs2102928 in skeletal muscle as the candidate variant affecting *ERBB2* expression, whereas rs1565922 in peripheral nerves was implicated in this current analysis. These findings are not mutually exclusive and point to this myasthenia gravis–related gene operating across multiple tissues.

Regardless of these subtle differences, the two studies hint at *CHRNB1* and *ERBB2* playing a prominent role in the pathobiology of myasthenia gravis. Given that *ERBB2* can modulate the expression of acetylcholine receptor subunits (3, 4), future studies should explore how the expression of these genes in nerves and skeletal muscle mediate the disease process. Our findings also have clinical implications as patients carrying the risk allele could have persistently lower expression of acetylcholine receptors, which may explain why some patients fail to enter remission (4). These observations suggest that new therapies modulating *CHRNB1* and *ERBB2* expression may benefit treatment-refractory patients. To demonstrate how genomic information can provide useful starting points to consider for therapeutic interventions, we performed in silico druggability testing on additional gene targets identified from the prioritization analysis of our genome-wide association study data (5). This approach identified 14 gene targets as potentially druggable, three of which have existing approved drugs or therapeutic agents in clinical testing (milatuzumab, forigerimod, and oprozomib; see Table 1) (6–8).

**Table 1.** List of prioritized druggable genes ranked according to their priority index (PI) scores

Drug	Current disease indication	Mechanism of action	Gene	PI rank	PI rating	Druggability	Seed gene
Milatuzumab	Orphan drug status for MM and CLL	HLA-DR antigens- associated invariant chain antagonist	*CD74*	2	4.59	1	N
Forigerimod	Phase III trial for SLE	Heat-shock cognate 71-kDa protein inhibitor	*HSPA8*	12	4.11	8	N
Oprozomib	Orphan drug status for MM and Waldenstrom's macroglobulinaemia	26S proteosome inhibitor	*PSMD4*	20	3.96	15	N
—	—	—	*HLA-DRA*	4	4.48	21	Y
—	—	—	*HLA-DQA1*	5	4.44	8	Y
—	—	—	*UBA52*	6	4.42	19	N
—	—	—	*HLA-DQB1*	17	4.05	8	N
—	—	—	*AP1G1*	18	4.00	1	N
—	—	—	*SH3GL2*	19	3.96	3	N
—	—	—	*ARF1*	22	3.93	4	N
—	—	—	*AP2B1*	23	3.92	2	N
—	—	—	*AP1B1*	25	3.87	1	N
—	—	—	*HLA-C*	28	3.76	1	Y
—	—	—	*AP1S3*	29	3.75	1	N

Drugs that are approved or in clinical testing are highlighted in yellow. MM, multiple myeloma; CLL, chronic lymphocytic leukemia; SLE, systemic lupus erythematosus; Seed gene indicates if the prioritized gene was used as a seed gene (yes [Y] or no [N]).

The work presented by Zhu et al. (1), together with our recent publication (2), demonstrates the value of genomic research in unraveling the pathogenesis of neurological diseases. Most notably, the insights provided by such large collaborative efforts pave the way for rational drug development and precision medicine efforts.
